# Modelling and measurements of distributions in an adult human phantom undergoing proton scanning beam radiotherapy: lung- and prostate-located tumours

**DOI:** 10.1007/s00411-021-00895-w

**Published:** 2021-03-02

**Authors:** Monika Puchalska

**Affiliations:** Radiation Physics, Technische Universität Wien, Stadionalle 2, 1020 Vienna, Austria

**Keywords:** Dosimetry, TLD, MC simulations, PHITS, Proton PBS, Secondary radiation

## Abstract

Proton radiotherapy has been shown to offer a significant dosimetric advantage in cancer patients, in comparison to conventional radiotherapy, with a decrease in dose to healthy tissue and organs at risk, because the bulk of the beam energy is deposited in the Bragg peak to be located within a tumour. However, it should be kept in mind that radiotherapy of cancer is still accompanied by adverse side effects, and a better understanding and improvement of radiotherapy can extend the life expectancy of patients following the treatment of malignant tumours. In this study, the dose distributions measured with thermoluminescent detectors (TLDs) inside a tissue-equivalent adult human phantom exposed for lung and prostate cancer using the modern proton beam scanning radiotherapy technique were compared. Since the TLD detection efficiency depends on the ionization density of the radiation to be detected, and since this efficiency is detector specific, four different types of TLDs were used to compare their response in the mixed radiation fields. Additionally, the dose distributions from two different cancer treatment modalities were compared using the selected detectors. The measured dose values were benchmarked against Monte Carlo simulations and available literature data. The results indicate an increase in the lateral dose with an increase of the primary proton energy. However, the radiation quality factor of the mixed radiation increases by 20% in the vicinity to the target for the lower initial proton energy, due to the production of secondary charged particles of low-energy and short range. For the cases presented here the MTS-N TLD detector seems to be the most optimal tool for dose measurements within the target volume, while the MCP-N TLD detector, due to an interplay of its enhanced thermal neutron response and decreased detection efficiency to highly ionising radiation, is a better choice for the out-of-field measurements. The pairs of MTS-6 and MTS-7 TLDs used also in this study allowed for a direct measurement of the neutron dose equivalent. Before it can be concluded that they offer an alternative to the time-consuming nuclear track detectors, however, more research is needed to unambiguously confirm whether this observation was just accidental or whether it only applies to certain cases. Since there is no universal detector, which would allow the determination of the dosimetric quantities relevant for risk estimation, this work expands the knowledge necessary to improve the quality of dosimetry data and might help scientists and clinicians in choosing the right tools to measure radiation doses in mixed radiation fields.

## Introduction

Particle beam cancer therapy is increasingly being used to treat various types of cancer (PTCOG 2019). Although proton radiotherapy is more targeted than the conventional radiotherapy using photons, it still affects healthy tissue surrounding the tumour to be irradiated. Currently, the most modern type of particle therapy, the pencil beam scanning (PBS) treatment, does not require any modifying devices in the beamline, because the narrow proton beams of changing energy used are steered by magnets and, thus, the extra radiation dose to the patient is, at least in the ideal case, only due to secondary particles produced only inside the patient’s body. However, PBS is a relatively new technique and still most of the out-of-field data are from the earlier technique where the proton beam energy is passively modulated. The group of cancer survivors who had received an intensive radiation treatment has considerably increased recently (PTCOG 2019). For this reason, it is very important to also consider the quality of life of the surviving patients in the decades following the treatment. Note that irradiation of healthy tissue has been recognized as one of the main reasons for side effects in radiotherapy that might, on the long run, induce a secondary malignancy (Newhauser and Durante 2011). Although the associated risk of secondary malignancies may be due to other risk factors, such as lifestyle and genetic predisposition often also responsible for first cancer, it has been proved that radiotherapy may also contribute to an increased cancer risk of cancer survivors (Newhauser and Durante 2011; Brenner and Hall 2008; Travis et al. 2003). It is noted that secondary neutrons, which are generated from protons undergoing nuclear interactions within the patient’s body can deposit a non-negligible dose in healthy tissue even far away from the tumour. Even if such out-of-field doses are rather small as compared to the target dose in the tumour, they may cause severe side effects if delivered to radiosensitive organs. As those side effects are strongly dependent on the radiation dose deposited outside the planned target volume, thorough investigations are needed to decrease out-of-field doses during particle therapy.

As lung cancer is the leading cause of cancer incidence worldwide (contributing 21.8% of the global number of new cases diagnosed in 2018), followed by colorectal and prostate cancers (which contributed 18.7 and 13.3% new cancer cases in 2018, respectively) (WHO 2020), the major attention in this study was paid to tumours located in lung and prostate. Proton radiotherapy is not always the first choice for lung and prostate cancer treatment. Actually, it is difficult to give a global standard for the treatment of those cancers as this depends on the stage of the cancer, and the indications vary by country and even by health care institution. Proton radiation therapy has proved to be an excellent option for low-risk prostate cancer as it delivers high control rates with very little toxicity (Zietman et al. 2010). However, the disadvantage of protons is the monetary cost to the individual insurer or to the patient. The primary factor driving the cost of proton delivery in prostate cancer is the number of required fractions. For instance, the standard modality for the early-stage prostate cancer treatment at Loma Linda University Medical Center (LLU) in the US is 44–45 fractions of 1.8 Gy (cobalt Gy equivalent) to a total of 79.2 to 81 Gy delivered over eight to nine weeks (Zietman et al. 2010). Recent results showed, however, that a hypo-fractionated proton therapy of 60 Gy in 20 fractions within four weeks was also safe and effective for early-stage prostate cancer patients (Slater et al. 2019). Even if for early-stage lung cancer patients, with no regional or distant metastases, the surgical resection is the standard treatment, many patients are not good candidates for surgery for medical reasons, such as poor pulmonary function, medical comorbidities or medically inoperable stages of cancer. LLU reported clinical experience in the early-stage non-small cell lung cancer with a dose of 70 Gy in ten fractions over two weeks (Bush et al. 2013). A total dose of 66 Gy in ten fractions was given to peripherally located tumours and 72.6 Gy in 22 fractions to centrally located tumours at the Proton Medical Research Center at Tsukuba, Japan (Nakayama et al. 2010). Moreover, the University of Texas MD Anderson Cancer Center has reported a modified less hypofractionated protocol with a total dose of 87.5 Gy at 2.5 Gy/fraction of proton therapy (Chang et al. 2017).

Risk estimation for the induction of secondary cancer is a complex task because direct measurements inside the human body is generally not possible, and therefore tissue-equivalent human phantoms are in use for measurements of any dose distributions. So far only two adult human phantom experiments characterizing the secondary radiation field produced by high-energy proton scanning beams can be found in the literature; one characterizing the out-of-field dose profiles from ions and photons while simulating a brain proton treatment (LaTessa et al. 2012), and the other one characterizing the neutron dose equivalents simulating a prostate proton treatment (Hälg et al. 2014).

Due to their small dimensions and lack of cabling thermoluminescent detectors (TLDs) are a key element in human phantom dosimetry studies (Stolarczyk et al. 2011; LaTessa et al. 2012; Knežević et al. 2018). Although there are many different types of TLDs, there is no universal detector able to determine the dose in mixed radiation fields, generated by interactions of primary radiation with the material of the beam guide system, therapy room and the body of the patient during particle radiotherapy. TLDs differ in sensitivity and dose linearity, and most importantly, in detection efficiency to radiation with different ionization density. Hence, the intercomparison of different types of TLDs in clinical conditions may lead to an improved quality of dosimetry data. In addition, realistic data on the response of these detectors are desirable for testing mathematical models of out-of-field doses.

The study presents experimental and Monte Carlo (MC) modelling results for proton PBS exposures in a tissue-equivalent adult human phantom irradiated for lung and prostate tumours. The main goal of the study was to investigate spatial changes in dose distributions after proton treatment targeted at different organs as well as to compare the response of the most common TLDs and the MC simulations.

## Materials and methods

### Dosimetry system

The measurements were performed with four types of TLDs all based on lithium fluoride (manufactured by RADCARD, former TLD Poland). The detectors were in the form of solid pellets of 4.5 mm diameter and 1 mm thickness. MTS-N (^nat^LiF:Mg,Ti) and MCP-N (^nat^LiF:Mg,Cu,P) detectors are the most common and cheapest thermoluminescent materials, containing a natural lithium composition. MTS-6 (^6^LiF:Mg,Ti) detectors are enriched with 95.6% of ^6^Li to increase the detector sensitivity for thermal neutrons. Conversely, MTS-7 (^7^LiF:Mg,Ti) detectors contain pure (99.9%) ^7^Li showing a greatly reduced response to thermal neutrons (Obryk et al. 2014). The dose linearity index, defined as the ratio between the luminescence signal per dose unit over the luminescence signal for a low dose lying in the linear response region of the detector (Horowitz, 2001; Olko et al. 2001), is equal to unity for LiF:Mg,Ti detectors for proton doses below ~ 3 Gy; above this value this detector shows a supralinear response (Sądel et al. 2015). In turn, LiF:Mg,Cu,P detectors show a linear proton dose response up to ~ 10 Gy, while above their response becomes sublinear (Sądel et al. 2015).

In the present study, MTS-N and MCP-N detectors constituted the majority of detectors (about 350 items of each type). Due to the small number of MTS-6 and MTS-7 detectors (only 15 items of each type), those detectors were located only at some positions outside the irradiated target. Prior to the irradiation, the LiF:Mg,Ti detectors were annealed for 1 h at 400 °C, followed by a cool-down to ambient temperature and an additional 2 h annealing step at 100 °C. In contrast, the LiF:Mg,Cu,P detectors were annealed for 10 min at 240 °C. To obtain the individual sensitivity factors of the TLDs, each detector was exposed to a ^90^Sr/^90^Y beta source and subsequently read out before the irradiations. The individual sensitivity factor for each detector is defined as the signal for that detector divided by the average signal of all detectors. All readings were performed with a RISO reader model DA-20, with 5 °C/sec heating rate from room temperature up to 270 °C for the LiF:Mg,Cu,P detectors and up to 360 °C for the LiF:Mg,Ti detectors. All detectors were pre-heated inside the reader for 20 s at 150 °C. The thermal annealing steps and readout parameters are summarized in Table 1. Four TLDs of each type were used for registration of the background dose accumulated from the time of annealing until the time of the dose readings after phantom exposure. Another four TLDs of each type were used for the reference calibration with a secondary-standard  γ-ray radiation source ^137^Cs. The measured signals were converted to units of absorbed dose in water. To consider possible signal fading effects, the calibration and background detectors were read out simultaneously with the experimental detectors. For the LiF:Mg,Ti detectors dose assessment was based on the height of the main peak. Regarding the LiF:Mg,Cu,P detectors, the short pre-heat inside the reader does not allow for full light signal conversion between the lower temperature peak 3 and the main peak 4 (Bilski 2006; Bilski and Puchalska 2010). Therefore, for the LiF:Mg,Cu,P detectors it was decided to quantify the glow curve signal through the integral of the counts in the glow curve region from 150 to 260 °C, integrating peak 3 and 4. This reduces the uncertainty of dose overestimation with time, caused by the different time between the irradiation and the readout. All detectors were read out within seven days after the irradiations.

**Table 1:** Evaluation parameters for TLDs used in this study

Detector	LiF:Mg,Ti	LiF:Mg,Cu,P
Pre-irradiation annealing		
Temperature (^o^C)	400 + 100	240
Time (min)	60 + 120	10
Preheat (inside the reader)		
Temperature (^o^C)	150	150
Time (s)	20	20
Readout		
Temperature (^o^C)	360	270
Heating rate (^o^C/s)	5	5

The mean organ dose was calculated by summing the dose measured for each internal detector position, normalized to the total number of detector positions within the organ *O* after subtraction of the mean background dose: ${\hat D_{\text{O}}}\, = \,\frac{1}{N}\mathop \sum \nolimits_{i = 1}^N {d_{\text{i}}}\, - \,{\hat d_{{\text{bg}}}}$, where *d*_i_ is the dose at position *i*, *N* is the total number of detector positions within organ *O* and ${\hat d_{{\text{bg}}}}$ is the mean background dose estimated from four non-exposed detectors.

### Experimental setup and irradiations

The measurements were performed inside an anthropomorphic tissue-equivalent ATOM phantom (type 701-D by CIRS) representing an adult male with a height of 173 cm and a mass of 73 kg. The phantom consists of 39, 25 mm thick, axial slices. Each slice includes a number of organ-pecific locations. Each of those locations has a 5 mm diameter hole containing a tissue-equivalent cylindrical plug for TLD placement. The TLDs were placed at the top of these vertical tissue-equivalent cylindrical plugs. The adult male phantom includes a total of 271 locations for TLD positioning. However, due to the limited number of TLDs available, not all positions could be filled. In total, 163 and 183 positions were filled with the MTS-N and the MCP-N detectors for the lung and the prostate exposures, respectively. Additionally, 15 positions in the out-of-field region were filled with the MTS-6 and the MTS-7 detectors for the lung treatment.

**Fig. 1:**
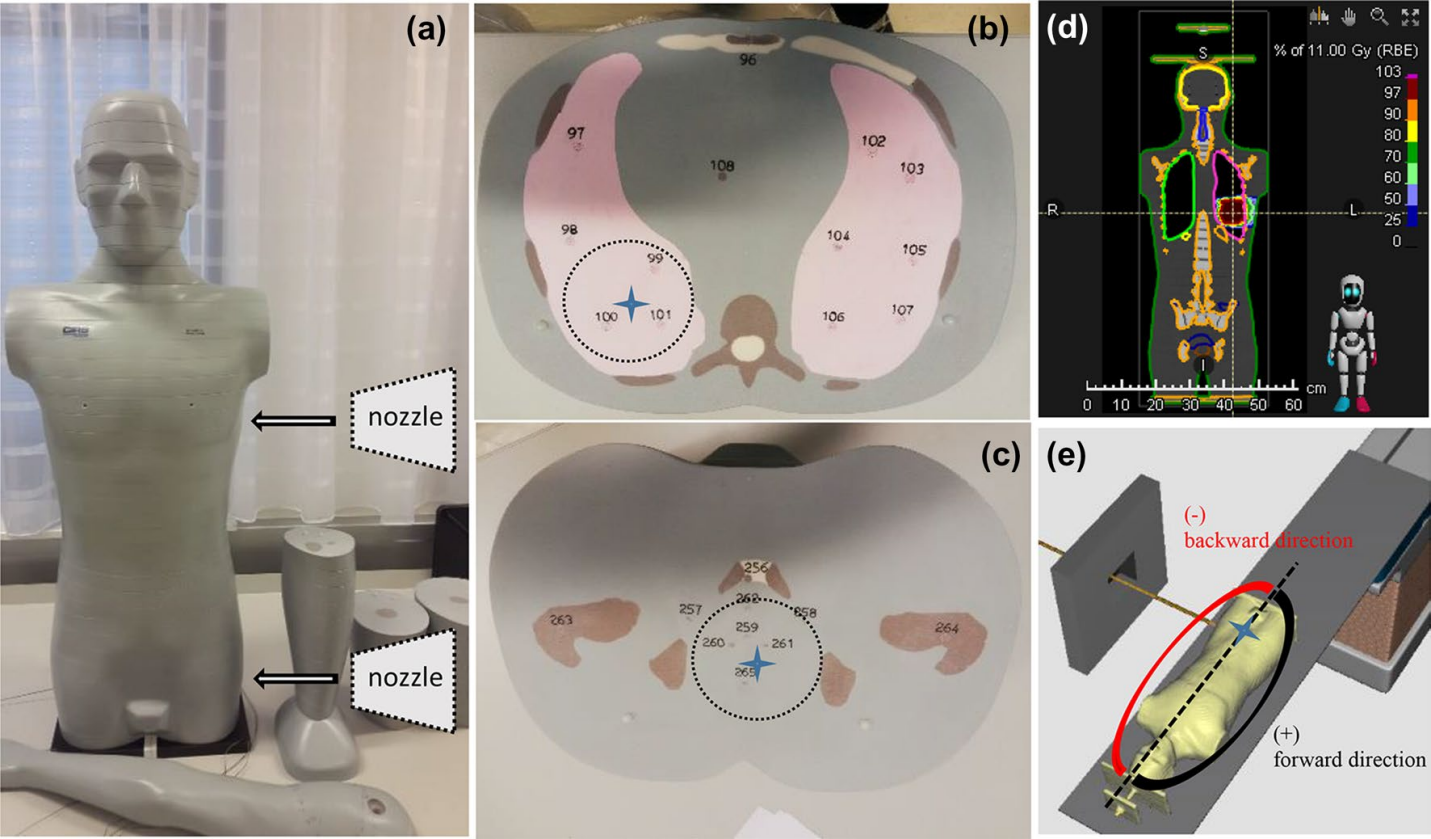
**a** Anthropomorphic ATOM phantom type 701-D. Arrows indicate the direction of the proton beam; **b**, **c** The slices #18 and #35 with the isocenters for the assumed lung and prostate tumours; **d**, **e** Planning with the treatment planning system (RayStation v.6.99). The blue cross marks the isocenter (**b**, **c**, **e**). The black dotted line in **e** indicates the laser passing the isocenter in the Inferior-Superior direction (sagittal plane) that divides the phantom into a positive (forward from the isocenter) and a negative (backward from the isocenter) direction. The same assumption was adopted for the lung case

Prior to irradiation, a CT scan of the phantom was performed and the required radiotherapy plans were prepared with a treatment planning system (TPS) RayStation v.6.99. The isocenter was located at the centre of the assumed tumour and the distance between the exit of the source and the isocenter was 66 cm. The long axis of the phantom was in both cases perpendicular to the beam direction (a fixed horizontal beam) and the source configuration was such that a uniform dose distribution was delivered to the target volumes by using scanning pencil beams with energies from 63 to 93 meV for the lung tumour and from 140 to 193 meV for the deeper located prostate tumour. To save time between entering the irradiation room, reorienting the phantom, and leaving the irradiation room, the treatment plans were not prepared according to the clinical protocols; instead, the beams were always delivered from only one direction (see Fig. 1). The irradiations were performed for target volumes of 212 and 176 cm^3^ for the assumed lung and prostate tumours, respectively. The measurements were carried out at the ion radiotherapy center MedAustron in Wiener Neustadt (Austria). This facility includes an irradiation room dedicated to research purposes, offering a fixed horizontal proton beam with PBS technology with an energy range from 60 to 800 meV.

To achieve a good measurement accuracy at the out-of-field positions, the assumed tumours (i.e., the target volumes) were irradiated with a total dose of 100 Gy, with a first fraction of 1 Gy followed by 9 Gy and then nine fractions of 10 Gy each. To avoid correction for supra- or sub-linearity and detector sensitivity loss after exposure to high doses (Bilski et al. 2008), after irradiating the target volume with a dose of 1 Gy the TLDs located directly in-beam were removed and only the TLDs located out-of-field remained for further exposure. Each exposure consisted of two rounds. In the first round the MTS-N detectors were positioned inside the phantom and irradiated. After reaching the total dose of 100 Gy to the target volume, all the MTS-N detectors were removed and in the second round of exposure the MCP-N detectors were positioned at exactly the same locations as those previously used for the MTS-N detectors. For the lung tumour exposure, besides those two main types of TLDs, also the MTS-7 and the MTS-6 detectors were irradiated alternately. Positioning of the phantom with a laser system allowed for high reproducibility in both rounds of exposures. The experimental setup and the TPS dose plans are shown in Fig. 1, where arrows indicate the beam directions.

### Simulations

Numerical simulations were performed with the general purpose MC particle transport code PHITS, v.3.10 (Sato et al. 2018), that has been already benchmarked for radiotherapy applications in several studies, showing a reasonable agreement with measurements and with other MC codes (e.g. Yang et al. 2017; Takada et al. 2018). The PHITS code is a multi-purpose 3D Monte Carlo code dealing with particle transport via continuous energy loss, collisions and decays via reaction models and cross-section data libraries. The intra-nuclear cascade model of Liège INCL4.6 (Boudard et al. 2013) was employed for the simulation of the dynamic stage of hadron-induced nuclear reactions. The quantum molecular dynamics model JQMD (Niita et al. 1995) was generally employed for nucleus-induced reactions. The evaporation and fission model GEM (Furihata 2000) was adopted for simulating the static stage for both hadron- and nucleus-induced reactions. The charged particle energy loss was calculated using the ATIMA code with a step size of 1 mm (ATIMA 2014). The cut-off energy for electrons/positrons, photons, and protons, was set to 1 × 10^−3^ meV, for neutrons to 1 × 10^−11^ meV, and for the other particles to 1 meV. Simulations were performed for 1 × 10^9^ particles.

Dose in the unit of Gy was scored in *T-deposit* tally, based on the mean energy deposited in a unit mass of the detector. The basic idea of *T-deposit* is to call the charged particle energy (e0) when entering the scoring region and again when leaving the scoring region (ef). Thus, the energy difference (e0–ef) gives the energy deposited in the considered region. The dose equivalent was calculated from the physical dose by multiplication with the *Q(L)* defined by (ICRP 1991). The relative biological effectiveness (RBE) was established based on the Microdosimetric Kinetic Model implemented in PHITS. The biological endpoint adopted in the estimation was a 10% surviving fraction for human salivary gland (HSG) tumor cells (Sato et al. 2009). Transport of low-energy neutrons was performed using the *Event Generator* mode, and the neutron dose was calculated with the function *counter*, which scores the energy deposited by their secondary charged particles.

The DICOM data obtained from the CT were converted to PHITS input format (voxel phantom) via the *DICOM2PHITS* routine. The relation between any CT value and material density is arranged in PHITS in a tabular form, based on (Schneider et al. 2000). The TLDs were simulated as liquid water. The voxel model of ATOM 701-D was created with 206 by 120 by 508 voxels. Resolution in the transverse plane was 2.34 mm per pixel and in the vertical plane 2 mm per pixel. The final voxel phantom consisted of 1.2 × 10^7^ voxels, while one TLD consisted of four voxels (4.68 × 4.68 × 2 mm^3^). A variance reduction technique was applied to increase the statistics at the measurement points (Niita et al. 2006).

### Simplified beam model

Proton beam delivery begins inside the accelerator (synchrotron @MedAustron) that accelerates protons to a prescribed energy and sends them to the beam transport system, and further to the treatment room. The final element in the beam delivery system is a nozzle. The nozzle not only delivers the beam to the patients but also monitors beam uniformity and alignment. While passing the transport system the initial energy of the protons slightly changes before reaching the isocenter. Therefore, a full-beam model including the dose and the spot position monitors is preferable for precise simulations. However, at MedAustron such data are confidential for users without clearance. Thus, based on the depth dose profiles measured with an ionization chamber (Bragg Peak chamber by PTW) in a water phantom and the PHITS simulations, a simplified beam model had to be developed, which was further used within this study. The model allows calculation of the energy loss of the protons inside the nozzle and provides the optimized proton beam energy and energy spread for further PHITS simulations. The energy is normally distributed. The spot size of a PBS has a certain size due to lateral scattering of the protons in the beam optics system. The size of the spot depends on the beam energy—with decreasing energy the spot size increases. Spot sizes, characterized as FWHM (full width at half maximum) of the lateral spread at the isocenter in air, for the key proton energies at MedAustron are summarized in Table 2.

**Table 2:** Full width at half maximum (FWHM) at the isocenter in air for proton beams measured with a LYNX detector at MedAustron based on an internal MedAustron report and simulated with the PHITS code

Proton energy (MeV)	FWHM-Lynx (mm)	FWHM-PHITS (mm)
62.4	21.1	19.8
97.4	14.0	13.2
148.2	10.0	9.5
198.0	8.3	7.9
252.7	6.9	7.2

## Results and discussion

### Simplified beam model

**Fig. 2:**
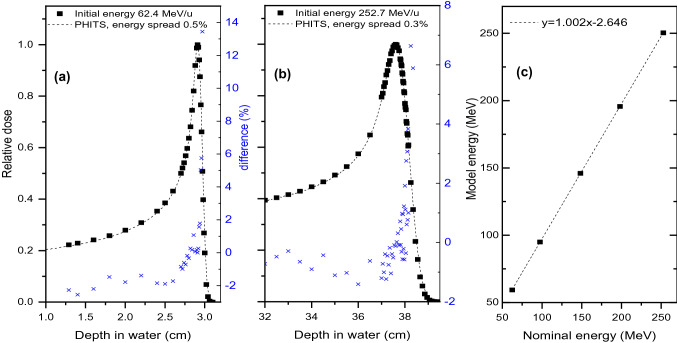
Measured and simulated depth dose profiles for an initial proton energy of **a** 62.4 meV and **b** 252.7 meV; **c** simplified beam model for the PHITS simulations. Blue symbols show the difference between the measured and the simulated depth dose profiles

The measured and the simulated dose profiles for protons with nominal energies of 62.4 and 252.7 meV are presented in Fig. 2a, b, while the simplified beam model, relating the nominal accelerator energy and the PHITS source energy, is shown in Fig. 2c. The energy loss due to the transport system elements in respect to the initial accelerated proton energy varies from 0.8 to 4%, for the highest and the lowest clinical energies at MedAustron, respectively. The difference between the measured and the simulated depth dose profiles is within 2% for the plateau region and 14% at R80 (depth of 80% of maximum dose on distal fall-off) for the lowest proton energy of 62.4 meV. The optimized spreads in proton energy were found to be 0.5 and 0.3% for the initial proton energy below and above 100 meV.

### Dose distributions

**Fig. 3:**
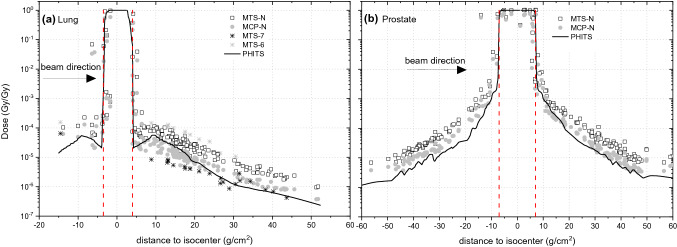
Absorbed dose distributions measured with TLDs (symbols) and simulated with PHITS (solid line) for tumours located in **a** lung and **b** prostate, both treated with proton pencil beam scanning (PBS). Data are normalized to the target treatment dose (Gy per treatment Gy). The combined relative measurement uncertainty for the TLDs ranged from 3% for doses higher than 2 mGy to 5% for doses below 2 mGy. The standard deviation for the results of the Monte Carlo simulations with PHITS was between 1% in the target region and about 30% at far distances from the isocenter. The target volumes are marked with dashed red lines and black arrows indicate the direction of the beam (see also Fig. 1); Note that the detector distance to the isocenter was calculated in 3D but is shown here in 1D

Figure 3a, b shows the deposited energy distributions measured with the four TLD types and the MC simulated doses inside the adult human phantom undergoing the lung and the prostate cancer treatments. The results are normalized to the target treatment dose (1 Gy for in-beam detectors and 100 Gy for detectors located outside the beam), referred here as Gy/Gy. The combined relative measurement uncertainty for TLDs ranged from 3% for doses higher than 2 mGy to 5% for doses below 2 mGy. The distance between the detector and the isocenter was calculated as the shortest connecting line in three-dimensions, considering voxel density. The data are organized such that they show the beam direction from the isocenter, located here in the middle of the coordinate system. The sagittal plane, passing the isocenter in the Inferior-Superior direction, divides the phantom into two sides, one in the backward (negative axis) and the other in the forward direction (positive axis) with respect to the beam direction from the isocenter.

The signal recorded by the detectors outside the target region derives from interactions of secondary particles, i.e. neutrons and photons, with the nuclei of the tissue-equivalent phantom material, producing avalanches of next-generation protons and neutrons, but also electrons, alpha particles and heavier nuclei. The dose values measured with different TLD types differ significantly. The lowest dose inside the adult phantom at about 50 cm to the isocenter for the lung exposure was 400 nGy/Gy was measured by the MCP-N detector, and 1 µGy/Gy by the MTS-N detector. For the prostate irradiation, the corresponding values were 2 and 8 µGy/Gy at a similar distance to the isocenter, for the MCP-N and MTS-N detectors, respectively. The ratio of the response of the MTS-N and the MCP-N detectors in the target volume was 1.2, for both treatments. The difference in response of those two types of TLDs is caused by the lower detection efficiency of the MCP-N detectors to radiation of high ionizing density (Bilski and Puchalska 2010). Due to the small ^6^Li content (7.6%) in both mentioned detectors an about three orders of magnitude greater thermal neutron sensitivity is observed in comparison to a detector containing pure ^7^Li (KAERI 2020). Consequently, an enhanced neutron dose response was observed when comparing the MCP-N and MTS-N output with that of the MTS-7 detector. The highest dose values were recorded with the MTS-6 detector, due to its very high sensitivity to thermal neutrons.

For better visualization, the simulated doses are shown with lines in Fig. 3, and these lines can be compared with the measured data. At the target volume, the simulations (and the TPS-planned dose) show a relatively good agreement (i.e., within a few percent) with the values measured with the MTS-N detectors, for both modalities. In contrast, the MCP-N detectors underestimate the dose in the target volume by about 20%. As dose gradients in PBS radiotherapy are very steep, the response of the detectors located at the edge of the target volume depends strongly on the size of the detector and the precision of positioning. Additionally, when a beam passes materials with different densities a slight misaligning of the beam might also cause a significant difference in detector response. As the distance of the detector position from the isocenter increased for the lung treatment, the ratio between the MTS-N measured dose and the simulated dose increased by factors 2 to 5. In contrast, the MCP-N detector underestimates the dose by 50% in close proximity to the target volume, while it tends to overestimate it up to a factor of 2, for greater distances. For the prostate tumour which is located in the middle of the phantom the ratio between MTS-N measured and simulated dose varies from 2 to 5 for a distance of −10 and −40 cm in front of the target volume, and then from 2 to 3 at corresponding distances behind the target volume. For the MCP-N detector the comparison between measured and simulated doses is much better, 1.1 to 1.5 times behind the target volume and 1.5 to 3 in front of the target volume. These differences are due to the fact that, although both types of TLDs show a similar content of ^6^Li, the MCP-N detector has a lower detection efficiency to radiation with high ionizing density (Bilski and Puchalska 2010). In turn, due to the significantly reduced sensitivity of the MTS-7 detector to thermal neutrons, this detector mainly underestimates the absorbed dose by up to a factor of 10, while the MTS-6 detector overestimates the absorbed dose on average by a factor of 7.

**Fig. 4:**
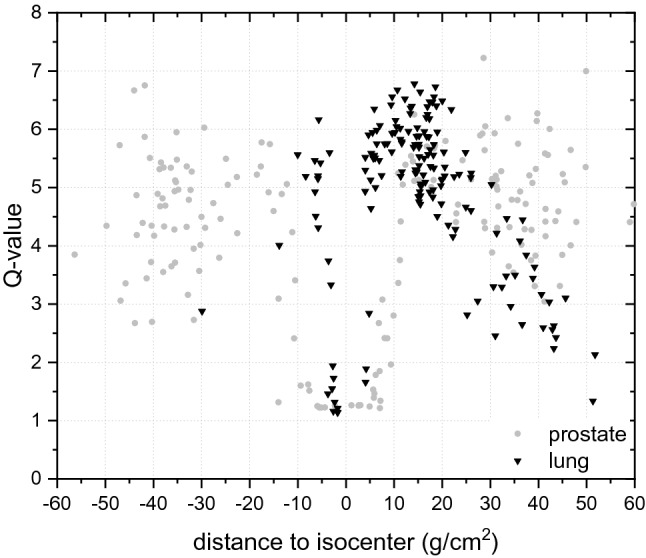
Quality factor Q simulated with PHITS on the basis of the Q(LET) relation recommended by (ICRP 1991). In the simulations, the standard deviation varied between 1% in the target volume and 30% at about 40 g/cm^2^ from the isocenter

The ionizing density is expressed in Fig. 4 via the radiation quality factor *Q*, calculated based on the linear energy transfer (LET), used in radiation protection to weight the absorbed dose with regard to its presumed biological effectiveness (ICRP 1991). For the lung exposure, a fast increase in *Q* is observed just behind the target volume, with a maximum value of 6.5 at a distance of 15 g/cm^2^, and a sharp decrease just after a few g/cm^2^. In contrast, for the prostate exposure the Q reaches a maximum value at a distance of 15 g/cm^2^ and keeps the elevated value oscillating around 5.

**Fig. 5:**
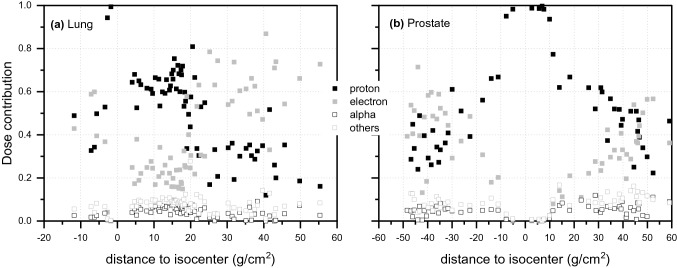
Charged particle contribution to the absorbed dose as a function of distance to the isocenter for **a** the lung and **b** the prostate exposure. In the simulations, the standard deviation varied between 1% in the target volume and 30% at about 40 g/cm^2^ from the isocenter

To explain these values of *Q*, the contribution of various particles to the absorbed dose was simulated (Fig. 5). It can be seen from the figure that for the lung treatment, in the forward direction from the isocenter up to a distance from the isocenter of about 20 g/cm^2^, the main dose contribution is due to secondary protons, while above 20 g/cm^2^ electrons dominate absorbed dose. The change of the material from lung tissue to the soft tissue at about 5 g/cm^2^ results in an enhanced secondary proton production, showing a local peak at about 15 g/cm^2^. For the prostate treatment, the secondary protons dominate the absorbed dose up to a distance from the isocenter of about +40 and  −25 g/cm^2^ in forward and backward direction from the isocenter, respectively. Their contribution decreases with distance to the isocenter, while electron production smoothly increases, with about 10% greater electron contribution to absorbed dose at far distances from the isocenter in the backward direction. Moreover, the absorbed dose contribution of alpha particles and heavier nuclei is slightly higher in the forward direction, due to interaction via (*n*,*p*) and (*n*,*α*) reactions with nuclides of low atomic number (Mares et al. 2016).

**Fig. 6:**
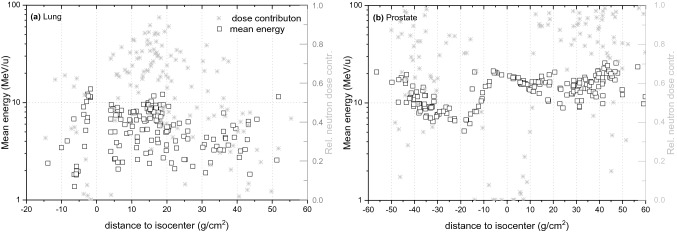
Mean energy and relative dose contribution of the secondary neutrons as calculated with PHITS code for tumours in the lung (left) and the prostate (right) after application of pencil beam scanning (PBS)

For prostate treatment, the symmetry in the measured doses, visible in Fig. 3b, does not translate into the symmetry observed for the simulated values. Meaning, the dose measured with the TLDs in the backward direction (negative distances to the isocenter, in Fig. 3b) appears slightly elevated compared to the simulated values. This elevation in the measured dose is related to a decrease in neutron energy in this direction, as shown in Fig. 6b, and an increase in relative contribution to neutron fluence of thermal neutrons reported in Table 3. Since both detector types, the MTS-N and the MCP-N, contain a few percent of ^6^Li the dose measured at negative distances to the isocenter is elevated, when compared to the simulated doses. The data presented in Table 3 show that high-energy neutrons (≥20 meV) are preferentially emitted in the forward direction. For the prostate, the difference between neutron fluence at forward and backward direction is about 20%. In contrast, for the lung only a few percent difference was observed because the energy of the primary protons in the beam was much lower and, consequently, the contribution of high-energy to total neutron fluence and the mean neutron energy was also higher than in the lung treatment plan. In general, the neutron spectra for the prostate treatment involve almost equally three main components, thermal (1 meV ≤ *E*< 0.4 eV), fast (100 keV ≤ *E* < 20 meV) and high-energy range neutrons (*E* ≥ 20 meV). In contrast, neutron spectra simulated for the lung treatment show a dominant component of thermal neutrons (~ 50%), followed by a component of fast neutrons (~ 40%). For both treatment modalities it was observed that slightly more thermal neutrons are produced in backward than forward direction and that the fast neutrons are emitted almost similarly. Note that in measurements of the stray neutron radiation field in scanning proton therapy using extended-range Bonner spheres at different positions around a paediatric phantom irradiated for a brain tumour and aligned along the beam axis, a large component of high-energy neutrons was also observed. These high-energy neutrons were preferentially emitted in forward direction while neutrons in the fast energy range were emitted isotropically from the place of origin (Mares et al. 2016).

**Table 3:** Relative contribution to neutron fluence, for various neutron energy regions in dependence on distance to the isocenter for the lung (A) and the prostate (B) irradiations

Lung													
Distance to isocenter				−20	−10	−5	0	5	10	20	30	40	50
High				0.02	0.03	0.03	0.14	0.09	0.12	0.09	0.06	0.08	0.16
Fast				0.31	0.32	0.32	0.53	0.40	0.36	0.35	0.36	0.30	0.37
Intermediate				0.12	0.13	0.17	0.11	0.14	0.11	0.11	0.10	0.11	0.12
Thermal				0.54	0.52	0.48	0.22	0.38	0.40	0.45	0.47	0.51	0.35
Mean energy (MeV)				2.5	2.6	2.6	8.3	5.5	6.8	5.3	4.3	4.8	6.4

Prostate													
Distance to isocenter	−50	−40	−30	−20	−10	−5	0	5	10	20	30	40	50
High	0.16	0.22	0.19	0.14	0.17	0.31	0.34	0.29	0.27	0.26	0.29	0.38	0.36
Fast	0.53	0.39	0.31	0.27	0.26	0.31	0.36	0.30	0.25	0.28	0.29	0.30	0.34
Intermediate	0.01	0.12	0.10	0.09	0.11	0.09	0.08	0.09	0.09	0.07	0.09	0.05	0.09
Thermal	0.30	0.27	0.41	0.49	0.46	0.28	0.22	0.32	0.39	0.39	0.33	0.27	0.25
Mean energy (MeV)	15.6	13.7	10.3	8.2	11.1	21.4	22.3	19.7	18.3	15.4	16.2	19.4	20.7

Thermal (1 meV ≥ *E* < 0.4 eV), intermediate (0.4 eV ≥ *E* <  100 keV), fast (100 keV ≥ *E* < 20 meV) and high (*E* ≥ 20 meV)

As expected, for further distances from the isocenter the absorbed dose was lower for both, the prostate and the lung treatment (Fig. 3a, b). Moreover, when comparing both treatments, a more pronounced dose reduction, i.e. by one order of magnitude, was observed for the lung as compared to the prostate treatment (Fig. 3a, b). This reduction in out-of-field dose is associated with the lower primary proton energies used for the lung tumour treatment as compared to that used for the prostate tumour treatment, but also with a three times lower density of lung tissue as compared to prostate tissue, leading to fewer secondary particles being.

### Organ doses

The organ doses measured with the MTS-N and the MCP-N detectors and simulated with PHITS are summarized for the lung treatment in Table 4 and for the prostate treatment in Table 5. The doses measured with the MTS-7 detectors are only fragmentary and did not allow to estimate organ doses. The mean organ distance to the isocenter and the number of detectors used for organ dose estimation in respect to all available positions in the organ are also included in the tables. The mean organ distance to the isocenter was calculated as an average value from individual points constituting a given organ.

**Table 4:** Measured and simulated mean organ doses per treatment gray after lung irradiation together with relevant RBE and *Q* values, mean organ distances to the isocenter and number of used TLDs as compared to all available positions

Lung	MTS-N (μGy/Gy)	MCP-N (μGy/Gy)	Filled/all positions	PHITS (μGy/Gy)	RBE	*Q*	Distance to isocenter (cm)
Heart	92 (10)	31 (3)	2/2	73 (20)	1.8 (0.1)	6.0 (0.1)	11 (1)
Oesophagus	94 (14)	33 (6)	3/3	28 (21)	1.8 (0.1)	6.0 (0.5)	13 (4)
Stomach	83 (65)	39 (41)	10/14	28 (29)	1.7 (0.2)	5.1 (0.8)	15 (5)
Kidneys	49 (32)	18 (12)	10/16	12 (10)	1.7 (0.1)	5.6 (0.7)	16 (8)
Liver	52 (19)	18 (7)	16/29	32 (33)	1.8 (0.1)	5.8 (0.6)	17 (5)
Lung (right)^a^	37 (9)	13 (3)	19/19	18 (5)	1.7 (0.1)	5.5 (0.5)	18 (2)
Breasts	138 (172)	96 (129)	2/2	61 (69)	1.7 (0.3)	4.9 (1.6)	18 (8)
Thyroid	23 (8)	8.4 (4)	6/6	6.4 (2.7)	1.7 (0.1)	5.3 (0.4)	22 (3)
Colon	8.6 (4.4)	3.2 (1.5)	8/16	1.7 (1.1)	1.4 (0.2)	3.7 (0.8)	30 (7)
Bladder	2.6 (0.5)	1.1 (0.2)	7/16	0.6 (0.3)	1.4 (0.2)	3.3 (0.8)	39 (3)
Brain	4.9 (2.2)	1.0 (0.3)	6/11	0.7 (0.3)	1.4 (0.2)	3.7 (1.4)	40 (4)
Testes	1.0 (0.1)	0.4 (0.1)	2/2	0.2 (0.1)	1.1 (0.1)	1.7 (0.6)	51 (1)

In brackets are one-sigma uncertainties

^a^The left lung received on average 155 (270) mGy/Gy

**Table 5:** Measured and simulated mean organ doses per treatment gray after the prostate irradiation together with the RBE and the *Q* values, the mean organ distance to the isocenter and number of used TLDs in respect to all available positions

Prostate	MTS-N (μGy/Gy)	MCP-N (μGy/Gy)	Filled/all positions	PHITS (μGy/Gy)	RBE	*Q*	Distance to isocenter (cm)
Bladder	4.6 × 10^5^ (4.7 × 10^5^*)*	3.2 × 10^5^ (3.4 × 10^5^*)*	16/16	3.7 × 10^5^ (4.7 × 10^5^*)*	1.2 (0.2)	2.0 (0.9)	6 (3)
Testes	523 (53)	229 (40)	2/2	129 (13)	1.6 (0.1)	4.9 (0.4)	12 (1)
Colon	2.1 × 10^4^ (7.9 × 10^4^*)*	1.9 × 10^4^ (7.3 × 10^4^*)*	16/16	6.3 × 10^4^ (2.5 × 10^5^*)*	1.6 (0.3)	4.5 (1.3)	15 (6)
Kidneys	71 (37)	29 (15)	15/16	20 (12)	1.6 (0.1)	4.6 (0.7)	29 (5)
Stomach	58 (40)	26 (16)	13/14	13 (7)	1.6 (0.2)	4.6 (1.0)	30 (5)
Liver	31 (11)	13 (5)	26/29	11 (6)	1.6 (0.2)	4.7 (1.0)	34 (4)
Heart	–	–	0/2	2.5 (0.8)	1.5 (0.1)	3.9 (0.1)	44 (2)
Lungs	14 (4)	6.5 (2.3)	24/36	3.7 (3.1)	1.5 (0.2)	4.3 (1.2)	47 (6)
Breasts	–	–	0/2	6.2 (6.8)	1.4 (0.1)	3.3 (1.0)	47 (1)
Oesophagus	16 (7)	6.6 (1.9)	3/3	2.8 (1.8)	1.6 (0.1)	4.7 (0.6)	50 (9)
Thyroid	–	–	0/6	0.5 (0.2)	1.6 (0.3)	5.4 (2.0)	60 (3)
Brain	–	–	0/11	0.3 (0.2)	1.3 (0.3)	3.3 (1.7)	79 (3)

In brackets one-sigma uncertainties

The highest organ doses were registered in the vicinity of the target volumes, e.g. in the bladder for the prostate treatment and in the heart for the lung treatment. Parts of the bladder and of the colon are located in the high-dose gradients at the edges of the target volumes, causing an increase in dose but also in mean dose uncertainty. Furthermore, for large organs or paired organs, e.g. breasts or kidneys, the spread in the distance between individual points in a given organ and the isocenter reaches 50%, causing also a large inhomogeneity in the dose to these organs. Since for epidemiological studies of secondary cancer induction the mean organ dose may not be an adequate quantity when the dose in the organ of interest is highly inhomogeneous (Schneider and Walsh 2017), in brackets in Tables 4 and 5 the statistical variations (one-sigma) are also provided. Due to the low statistics for organs far away from the target volume, or because of the small number of measuring points, the accuracy of doses given in these tables for certain organs is limited.

As already mentioned, the content of ^6^Li in the MTS-N and MCP-N detectors causes an increase in measured neutron dose. However, in the MCP-N detectors this effect is partly compensated by a smaller detection efficiency for protons. Therefore, the mean organ doses measured with the MCP-N detectors (Tables 4 and 5) appear to agree best with the simulated mean organ doses. The MCP-N detectors tend to overestimate the mean organ dose by about a factor of 2. However, for organs located near the target volume for the lung treatment, e.g. the liver or the heart, the mean organ doses are highly underestimated (Table 4) as the radiation density is elevated in this region. The mean organ dose measured with the MTS-N detectors is always conservative (i.e., higher) when compared to simulated doses, with an average factor of 4 and 3 for the prostate and the lung treatments, respectively.

The available literature on dose measurements in proton radiotherapy using a human phantom shows that the most studied cases involve tumours located in the head and that doses to other organs were not given (La Tessa et al. 2012), or where data on the mean organ distance to the target volume were not given (Stolarczyk et al. 2011). Furthermore, a comparison of the results obtained in the present study could not be done with those obtained by (Knežević et al. 2018) because the phantom used by these authors was of another size.

Based on simulated absorbed doses and the quality factor Q (see Table 4), the dose equivalent to the heart, after a typical total dose of 70 Gy to the target volume for lung treatment, was estimated here to be about 30 mSv. This value is below the limit of 100 mSv which was recognized by the International Commission on Radiological Protection as potentially provoking non-cancer diseases (ICRP 2007).

### Neutron dose equivalent

**Fig. 7:**
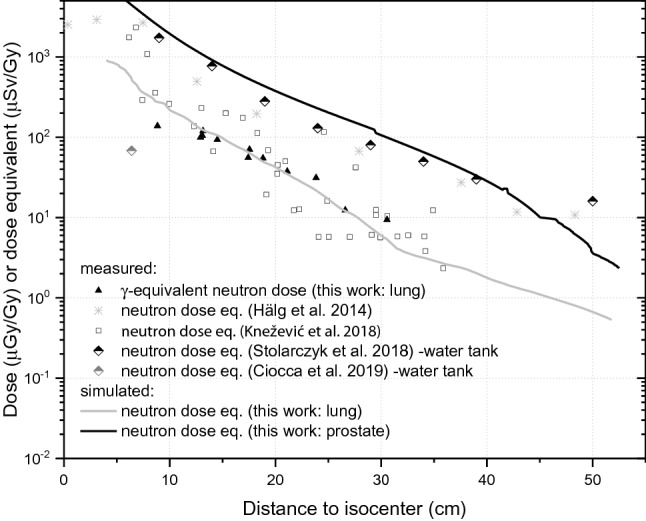
Neutron dose and neutron dose equivalent measured and simulated in a human phantom irradiated for the lung tumour treatment with proton PBS beams. Literature data are also included in the comparison

In Fig. 7 the measured and the simulated neutron dose equivalent (*H)*, in the unit of μSv/Gy, is plotted together with some literature data. The MTS-6 and MTS-7 detectors were calibrated in terms of γ-ray dose, so the difference between their readings gives the γ-equivalent neutron dose that is an indicator of thermal neutron fluence. In principle, this could not be directly related to the mean neutron dose, as the spectrum in proton treatment is attributed predominantly to fast and high-energy neutrons. However, a conversion from the γ-equivalent neutron dose to *H* is possible via comparable measurements with e.g. nuclear track detectors or via simulations (Takam et al. 2009). Such a conversion factor was recently elaborated by Knežević et al. (2018) based on the net MTS-6–MTS-7 signal and the *H* measured by the nuclear track detectors inside a child phantom irradiated with proton PBS at energies between 70 and 140 meV. The conversion factor was found to be equal to unity. Based on the simulated values of the *H* and the dose difference between the MTS-6 and the MTS-7, presented in Fig. 7, the mean conversion factor from the γ-equivalent neutron dose to *H* was found in the present study to be 1.03 ± 0.32, showing a very good agreement with the results by (Knežević et al. 2018). However, the values of the conversion coefficient are not always equal to unity as was reported in (Stolarczyk et al. 2018).

In one of the recent papers on secondary radiation in PBS proton radiotherapy the *H* was measured using track etch detectors positioned along the central axis of an anthropomorphic RANDO phantom while simulating a prostate proton treatment (Hälg et al. 2014). The data obtained show that for a close distance of 10 cm from the isocenter, *H* was about 2 mSv/Gy and for far distances of about 30 cm *H* was about 70 µSv/Gy. In the present study, the simulated values of *H* for the prostate treatment were about 2 mSv/Gy and about 100 µSv/Gy for the distance of 10 and 30 cm from the isocenter, respectively. Thus, the simulated results show a very good agreement with independently measured data for prostate treatment.

Recently, a review article that summarizes different aspects of neutron dose in proton therapy was published by (Hälg and Schneider 2020). It appears that only a few of the scientific papers reviewed were dedicated to proton PBS, and none of them deal with irradiations of an adult human phantom. Hence, the comparison of the results in the present study was extended to the data measured in water phantoms. The data for the bubble detectors placed inside a water tank irradiated with an energy range between 62.7 and 89.8 meV used for eye treatments (Ciocca et al. 2019) suggest that *H* is about ten times lower than those obtained in the present study for a similar primary proton energy used for the lung treatment. Unexpectedly, for a brain tumor of 65 cm^3^ volume located inside a paediatric phantom irradiated with proton PBS of energies in the range 70–140 meV (Knežević et al. 2018), the *H* measured with track-etched detectors showed similar values as the ones simulated in the present study for the lung tumour with a target volume twice as large and much lower primary proton energy. In addition, the *H* measured with track-etched detectors by Stolarczyk et al. (2018), for a 1000 cm^3^ target volume located inside a water tank irradiated with proton PBS at an energy range of 120–170 meV, is comparable to the *H* measured with the same detector type in an adult human phantom treated for prostate cancer (Hälg et al. 2014), and to the *H* simulated in the present study for the prostate tumour, despite a five times bigger target volume and slightly lower primary proton energy. The above comparisons suggest that a specific combination of the primary proton energy and the volume of the irradiated target are responsible for the different, or similar, values of *H* measured in proton radiotherapy by various research groups. To more accurately determine the effect of the target volume and the impact of the initial proton energy on *H*, further studies are needed, e.g. inside the water tank phantom used in (Stolarczyk et al. 2018), extended to different target volumes and to different energy ranges of the primary proton beam.

## Conclusions

The present study compares results of 3D measurements and simulations of doses inside an anthropomorphic phantom, representing an adult male exposed for treatment of lung and prostate tumours with modern proton PBS radiotherapy. The comparison of the obtained lateral dose profiles demonstrated one order of magnitude lower dose levels for the lung exposures than for the prostate exposures, due to the lower initial energy of the primary protons in the beam. However, the produced secondary charged particles which show a higher ionization density, and therefore lower kinetic energy and shorter range, are responsible for a 20% increase in radiation quality in close proximity to the lung tumour, as compared to the prostate tumour case. In turn, for the prostate treatment at distances greater than about 25 cm from the target volume, the radiation quality remains at a higher level than for the lung treatment.

Comparison of the doses obtained with different TLD detectors used in the present study and with those simulated showed that for the present study the MCP-N detector is the best choice for out-of-field measurements, due to an interplay of enhanced thermal neutron response linked to the ^6^Li content of this type of detector and its decreased detection efficiency to high-LET radiation. In contrast, at the target volume the detection efficiency of the MTS-N detector seemed to be a perfect match for cases presented here. In general, doses are the most overestimated and the most underestimated by the MTS-6 and the MTS-7 detectors, respectively.

The dose equivalent to the heart for the left lung treatment was found below the limit recognized as potentially provoking non-cancer diseases. However, it should be kept in mind that for low doses it is generally unclear whether or not any damage to an organ will occur because a threshold level for stochastic effects does not exist and damage may occur at any radiation dose. Therefore, even if proton PBS represents an optimal technique for reducing out-of-field doses, attention should be paid to the tissue directly behind the irradiated target volume and to close-by vital organs, such as the heart or the lungs, to prevent any late toxicity in a population already predisposed to lung and cardiac injury.

The present simulations show that the component of high-energy neutrons is preferentially emitted in the forward direction while the fast neutrons are emitted quite isotropically. A slight increase in thermal neutron fluence in a backward direction from the isocenter causes dose enhancement for detectors that include even a small amount of ^6^Li isotopes that are highly sensitive to the presence of thermal neutrons.

The calibration factor used to calculate the neutron dose equivalent from the γ-equivalent neutron dose based on pairs of MTS-6 and MTS-7 detectors was found to be about unity. This agrees very well with previous findings. However, more investigations must be performed to unequivocally confirm whether or not this observation is just accidental. It is noted that any comparison of neutron measurements from different studies is, unfortunately, not a straight forward task, because many factors affect the results: in any case, however, a clear dependence of neutron dose equivalent on the primary proton energy and the volume of the irradiated target is visible.
